# Two-photon GCaMP6f imaging of infrared neural stimulation evoked calcium signals in mouse cortical neurons in vivo

**DOI:** 10.1038/s41598-021-89163-x

**Published:** 2021-05-07

**Authors:** Attila Kaszas, Gergely Szalay, Andrea Slézia, Alexandra Bojdán, Ivo Vanzetta, Balázs Hangya, Balázs Rózsa, Rodney O’Connor, David Moreau

**Affiliations:** 1 Mines Saint-Etienne, Centre CMP, Département BEL, F - 13541 Gardanne, France; 2 Institut de Neurosciences de la Timone, CNRS UMR 7289 & Aix-Marseille Université, 13005 Marseille, France; 3 Laboratory of 3D Functional Network and Dendritic Imaging, Institute of Experimental Medicine, Budapest, 1083 Hungary; 4 Lendület Laboratory of Systems Neuroscience, Institute of Experimental Medicine, Budapest, 1083 Hungary; 5 Two-Photon Laboratory, Faculty of Information Technology, Pázmány Péter Catholic University, Budapest, 1083 Hungary

**Keywords:** Ca2+ imaging, Multiphoton microscopy, Biological fluorescence, Cellular imaging, Imaging

## Abstract

Infrared neural stimulation is a promising tool for stimulating the brain because it can be used to excite with high spatial precision without the need of delivering or inserting any exogenous agent into the tissue. Very few studies have explored its use in the brain, as most investigations have focused on sensory or motor nerve stimulation. Using intravital calcium imaging with the genetically encoded calcium indicator GCaMP6f, here we show that the application of infrared neural stimulation induces intracellular calcium signals in Layer 2/3 neurons in mouse cortex in vivo. The number of neurons exhibiting infrared-induced calcium response as well as the amplitude of those signals are shown to be both increasing with the energy density applied. By studying as well the spatial extent of the stimulation, we show that reproducibility of the stimulation is achieved mainly in the central part of the infrared beam path. Stimulating in vivo at such a degree of precision and without any exogenous chromophores enables multiple applications, from mapping the brain’s connectome to applications in systems neuroscience and the development of new therapeutic tools for investigating the pathological brain.

## Introduction

Neurostimulation tools have been widely used in fundamental neuroscience and often lead to therapeutic applications^1^, where an external energy source must be applied to influence the activity of neurons. In the majority of research and clinical applications, the delivery of current pulses is used to stimulate neurons due to their electrical excitability. Yet, despite the numerous clinical methods for functional neurostimulation, cortical mapping and neuroprosthetics, electrical neurostimulation suffers from several limitations including poor spatial selectivity, high invasiveness, placement constraints and the generation of electrophysiological recording artefacts^2^.

Optical stimulation of the brain allows bypassing many of the existing limitations of classical electrical neurostimulation. An increasingly popular means for the optical stimulation of neurons is optogenetics^3^. This method is less invasive, artefact free (at least in normal energy ranges) and has the ability to reach high spatial selectivity when used in optimized conditions; however, it requires the genetic manipulation of neurons in order for them to express light sensitive opsin-based ion channels. This is usually achieved through viral transfections, which—despite their good controllability and the advantage of offering cell type specificity through the use of a promoter—, strongly limits applicability in humans. This is not the case for infrared neural stimulation (INS), an alternative to optogenetics, which has gained more and more interest in the last decade^4–8^, as it has the same advantages as optogenetics, yet without requiring any genetic manipulation. Indeed, in this case, the infrared radiation (typically around 1470 nm, 1860 nm or 2120 nm)^9^ interacts directly with the biological tissue without the need of any exogenous agents, thus offering numerous advantages for translation to clinical applications. Although INS is known to not be cell type specific, it has been shown recently that by applying proper parameters, it is possible to selectively and reversibly modulate small-diameter axons activity at lower radiant exposures rather than large-diameter axons^10^.

INS is known to trigger neural activity through a photothermal mechanism^11^. The water present in tissue absorbs infrared light, leading to a local increase of temperature. Thereafter, several hypotheses have been proposed for the subsequent biological effect of infrared-induced heating. The involvement and subsequent activation of temperature sensitive ion channels TRPV1 and TRPV4 has been demonstrated and shown to play a role in the initiation of the infrared-induced neural response^12,13^. Shapiro and colleagues proposed a more general electrostatic mechanism, where the INS depolarizes the lipid membrane bilayer through a change of the membrane capacitance induced by the temperature gradient^14,15^. Plasma membrane nanoporation has also been proposed^16^. Intracellular effects have also been demonstrated, particularly an increase of intracellular calcium ion concentration, originating from mitochondrial stores in cardiac cells^17^ or from the endoplasmic reticulum, via activation of the phospholipase C and the inositol trisphosphate (IP_3_) signaling pathway in neural and glial cells^18^, chinese hamster ovary cells, neuroblastoma-glioma cells^19^, and in cultured spiral ganglion neurons^20^. More recently, the role of the fast activation of voltage-dependent potassium ion channels in unmyelinated axons in the frame of infrared inhibition has been suggested in both modeling and experimental studies^21,22^.

Up to now, INS has been extensively studied in the peripheral nervous system with successful applications in the auditory system^23,24^, in chronic neurostimulation applications^25^, in the vestibular and facial nerves^26–28^, and as a method for cardiac pacing^29^. The first application of INS in the central nervous system (CNS) was demonstrated by Cayce and colleagues in 2010 in thalamocortical brain slices^30^. In the few studies carried out in vivo in the CNS, INS was shown to have an inhibitory effect in rat somatosensory cortex^31^, and an excitatory effect in non-human primates in the primary visual cortex^32^. In both of those cases, neural activity was assessed through optical intrinsic imaging or single unit electrophysiological recordings. More recently, INS has been used in combination with fMRI and was demonstrated to be a useful tool to map mesoscale brain connectomes in both cat and squirrel monkey^33^. To the best of our knowledge, only one study has used calcium imaging in vivo with simultaneous infrared brain stimulation in rodents^34^. In that one study, the synthetic indicator Oregon Green 488 BAPTA-1 AM was used to transiently label cortical astrocytes and neurons. Thereafter, infrared pulses of 250 µs were delivered at a frequency of 200 Hz for 500 ms and subsequent intracellular calcium fluorescence responses were observed in cortical astrocytes and apical dendrites on the brain surface. Deep neuronal tissue infrared modulation was also recently shown in rat neocortex and hippocampus, through the use of an implantable silicon microdevice enabling infrared delivery with an embedded waveguide and neuronal activity measurement with platinum microelectrodes^35^.

Here we used two-photon microscopy in vivo to test whether INS can be used to evoke intracellular calcium signals, in the mouse cortex in vivo*,* with a cellular resolution. Our first finding is that trains of short infrared pulses (250 µs delivered at 200 Hz for a duration of 500 ms as in^34^) initiate calcium signals in cortical neurons down to Layer 2/3 of mice expressing GCaMP6f. Second, we show that above a given threshold, the number of cells exhibiting calcium fluorescence responses increases with the amount of energy delivered locally to the cortex, and we investigated the spatial extent and pattern of the activation. Over the four different energy densities applied, we found that 87.5% of the cells located in the center of the beam were responsive at least to one energy density applied, compared to 50.9% for outside of the central part of the beam path. Also, the cells which exhibited infrared-induced calcium signals for multiple energy densities were found to be located mainly in the central part of the beam path. Third, we show that also the cells’ response amplitude depends on the amount of energy that is locally delivered. Importantly, we found no evidence for brain tissue damage, even at the highest INS intensity levels, as assessed using GCaMP6f fluorescence baseline stability as an in-vivo criterion, together with post-mortem histological analysis. Finally, our results show that two-photon microscopy is not only perfectly compatible with INS, but also offers the possibility of investigating infrared-induced calcium signals in multiple cells simultaneously with single-cell, and, in principle, sub-cellular resolution.

## Results

### Positioning of the fiber and the mouse for imaging

In order to perform cortical infrared stimulation simultaneously with two-photon imaging, we positioned an optical fiber to deliver infrared radiation at a fixed angle with respect to the surface of the brain. Most in vivo two-photon microscopes use water immersion objectives with high numerical apertures, and working distances between 2 and 3 mm when external access to the sample is needed (e.g. stimulation electrode, patch pipette, local drug perfusion with pipette, etc.). With such objectives, mechanical constraints limit the positioning of an external optical fiber. Indeed, when the optical axis was perpendicular to the cortical surface, the best (i.e. steepest) approach angle we could achieve with a bare fiber was about 26° in respect to the optical axis, which was impractical because the fiber adhered to either the microscope objective or to the cortex, hindering precise positioning.

**Figure 1:**
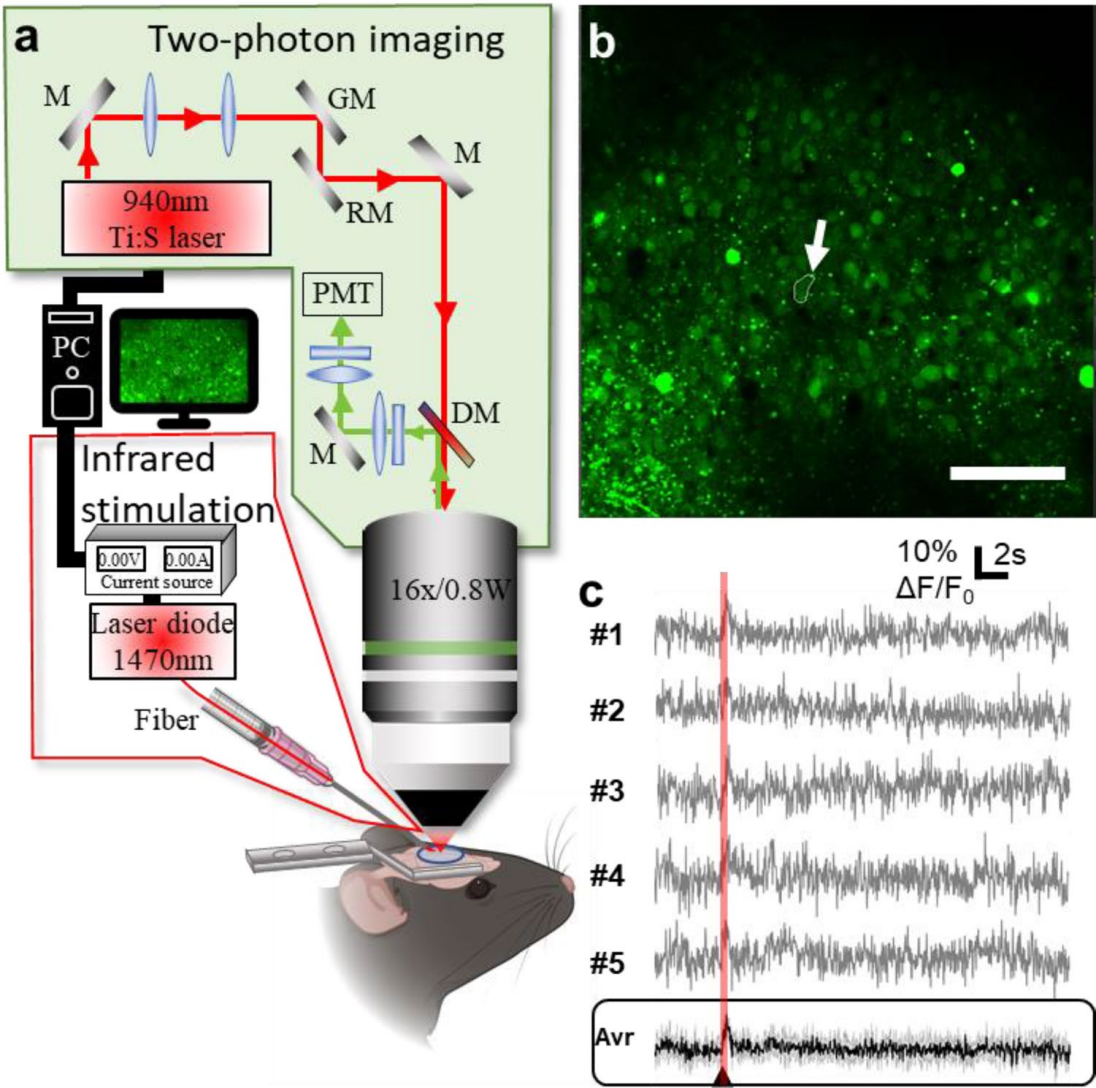
(**a**) Experimental setup for combined two-photon imaging and infrared stimulation. (**b**) Two-photon image of the area recorded showing cells targeted by infrared radiation. The arrow indicates the cell whose traces are shown in (**c**) Scale bar: 100 µm. (**c**) Representative example of infrared-induced intracellular calcium change in a single cell over 5 consecutive trials (0.58 J cm^−2^). *Bottom panel*, average of the 5 recordings with 5 consecutive identical stimuli. In black: the average of the repetition of the five recordings reproduced with the same infrared exposure parameters; in grey: standard deviation. The arrowhead marks the beginning of the infrared exposure (figure created from Powerpoint 2016, MESc software and Matlab R2018b).

In order to gain rigidity and avoid capillary attraction (i.e. to the immersion liquid, to the cortex, to the objective), we placed the fiber in a custom-built guide cannula. However, the cannula required more space than the bare fiber when its tip was advanced towards the mouse head; this was obtained by tilting the animal with respect to the optical axis of about 22°, up to the point where the cannula could be adequately advanced towards the cortex using a micromanipulator (see Supplementary Movie S1 for 3D representation of positioning). Figure 1 shows a scheme of the experimental setup focused on the mouse under the microscope, where the angle of the mouse head was tilted at 13° with respect to the optical axis, giving an incident angle of the fiber with respect to the brain surface and the optical window of about 35°.

### Infrared-induced intracellular calcium concentration signal in single cells

Our first goal was to check whether pulsed infrared light trains were able to evoke intracellular calcium signals in Layer 2/3 neurons in mouse cortex in vivo (Fig. 1). We thus acquired two-photon calcium fluorescence images at 30.25 frames per second for a duration of 30 s from GCaMP6f labeled mouse cortices, using neuron-specific viruses (see “Methods”). In order to obtain a fluorescence baseline, we applied infrared neural stimulation through an optical fiber (positioned towards the cortex as described previously) 5 s after the beginning of the recordings. The stimulation protocol consisted of 100 pulses of 250 μs repeated at 200 Hz, as used previously in the literature^31^. We repeated this stimulation protocol 5 times for each energy density in a recording loop, in order to assess the reproducibility of the response on the one hand, and, on the other hand, to increase the signal-to-noise ratio of the observed effect for each single cell by taking the average (which was used for all the following data analysis, unless specified otherwise). Figure 1b shows the observed cortical area of interest for the infrared stimulation recordings. The depth of the cells imaged in the field of view was determined from the z-stack and found to vary from 96 μm till 255 μm under the brain surface as a result of the tilting of the animal head, corresponding to the location of Layer 2/3 neurons (see Supplemental Movie S1). As shown by Fig. 1c, a reproducible increase in intensity of GCaMP6f fluorescence was observed in single cells following infrared exposure, in this case shown for pulse energy density of 0.58 J cm^−2^, calculated at the brain surface (see further down for a systematical discussion of the effects of delivered energy density).

**Figure 2:**
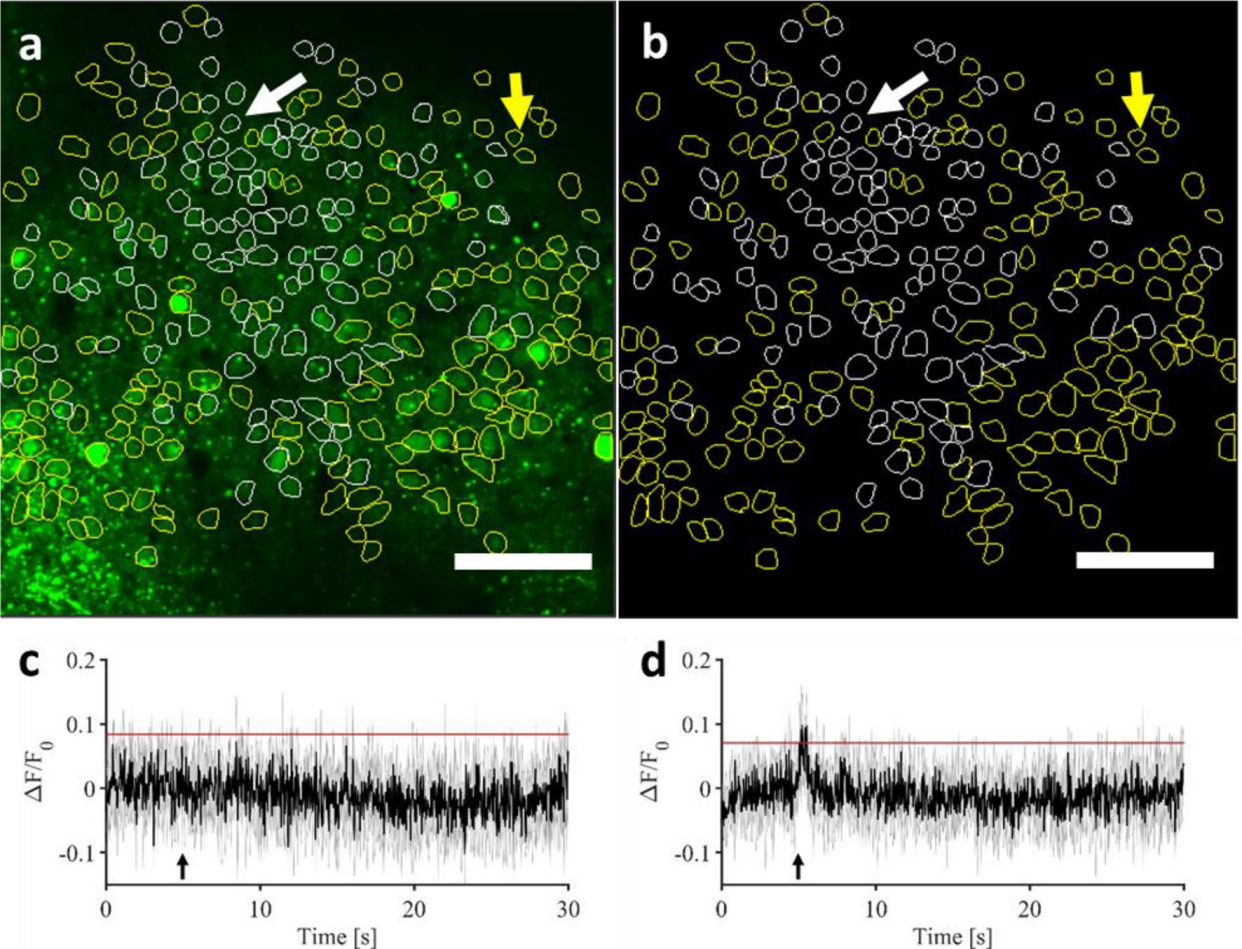
(**a**,**b**) Representative two-photon field of view of a single mouse visual cortex showing responsive identified cells (white contours) and non-responsive cells (yellow contours), with adjusted lookup table in (**b**). for better visualization. The arrows denote the two cells taken as example in (**c**) and (**d**) Scale bar: 100 µm. (**c**,**d**) Examples of 5 recordings average GCaMP6f fluorescence time course in a single cell not responding and responding, respectively, to the infrared exposure (marked, respectively, by white and yellow arrows on **a** and **b**). Black line: average of the repetition of the five recordings reproduced with the same infrared exposure parameters (0.70 J cm^−2^); grey: standard deviation. The black arrow marks the beginning of the infrared exposure. Red lines represent the threshold used in all figures and data analysis to identify responding and not responding cells (see “Methods” for thresholds determination) (figure created from MESc software and Matlab R2018b).

In order to gain a better view of the whole image field, all of the cells that could be identified (294 cells in total) as regions of interest (ROIs) were selected and the average fluorescence traces were calculated over the 5 stimulation trials for each cell. Figure 2 shows the two-photon field of view with all the regions of interest considered (in white the responsive cells, in yellow the non-responsive cells). Panels c and d show the evolution of two different types of signals over time that could be observed across all cells: both cells responding to the infrared exposure by an increase in the GCaMP6f fluorescence, and those that didn’t.

### Energy density affects infrared-induced response

Given the capability of pulsed infrared light trains to evoke intracellular calcium ion concentration changes in single cells, we systematically tested different levels of infrared power (and thus the delivered energy density), in order to assess stimulation and damage thresholds, as well as the relationship between the amplitude of the GCaMP6f fluorescence change with the energy density delivered. We investigated four different pulse energy densities (calculated at the surface of the brain): 0.35 J cm^−2^, 0.47 J cm^−2^, 0.58 J cm^−2^ and 0.70 J cm^−2^, always taking 5 recordings in a loop for each energy level, as explained previously.

**Figure 3:**
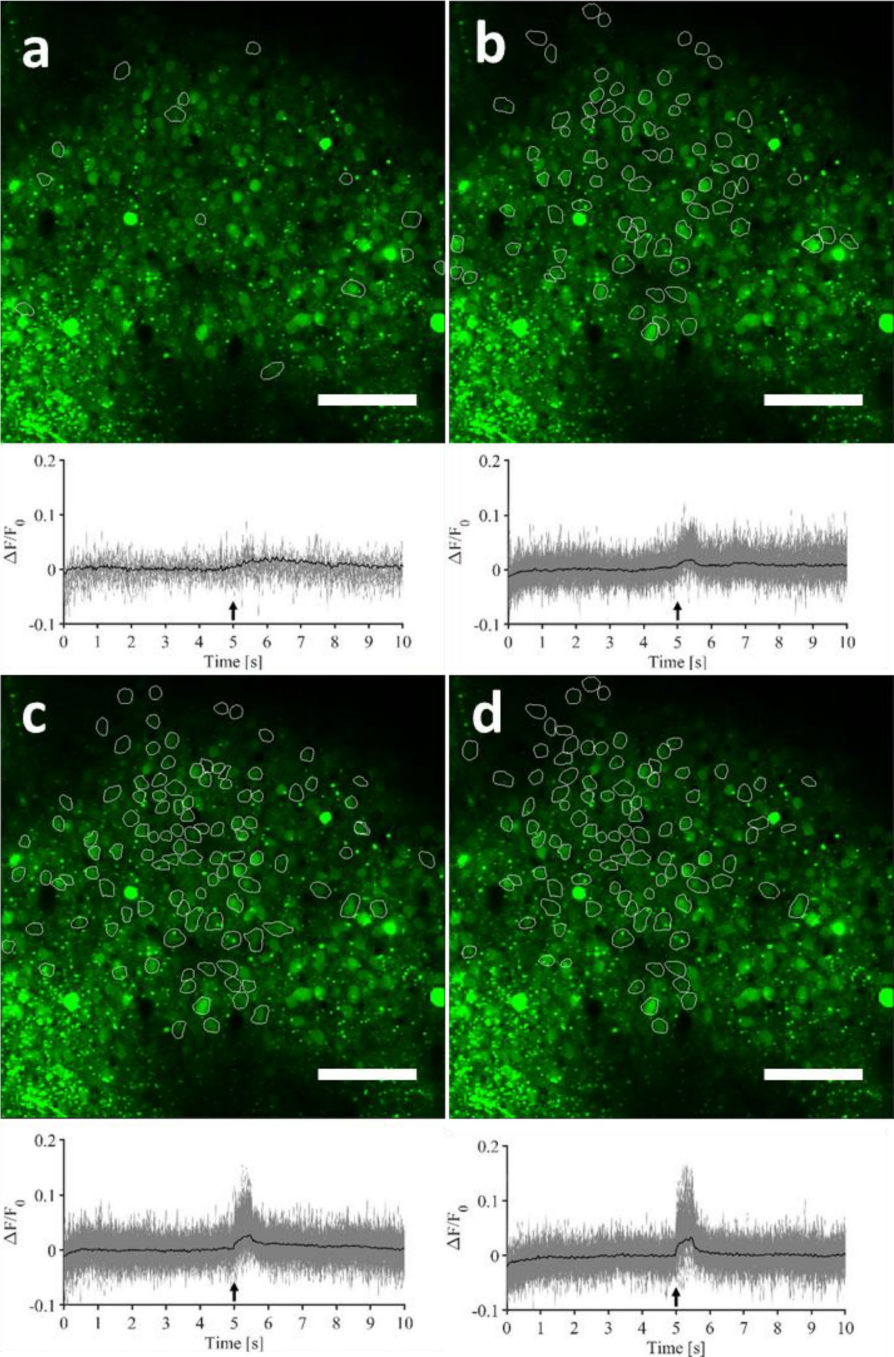
Representative two-photon field-of-view of a single mouse visual cortical area showing identified responsive cells, as well as their individual GCaMP6f fluorescence time courses (grey—average over 5 repetitions) and the grand-average GCaMP6f fluorescence time course, i.e. the 5-trial average, averaged over all the responding cells (black). (**a**) 0.35 J cm^−2^. (**b**) 0.47 J cm^−2^. (**c**) 0.58 J cm^−2^, and (**d**) 0.70 J cm^−2^. Scale bar: 100 µm. For the criterion used to class a cell as responsive or not, see Fig. 2c,d and “Methods”. This figure is modified from^36^, as the pictures have already been published in early work in a proceeding of SPIE Photonics West (figure created from MESc software and Matlab R2018b).

We found that increasing the delivered pulse energy induced a general increase in the number of responding cells, as shown by the increasing number of outlines in the images and traces in the graphs, when proceeding from Fig. 3a towards 3d.

**Figure 4:**
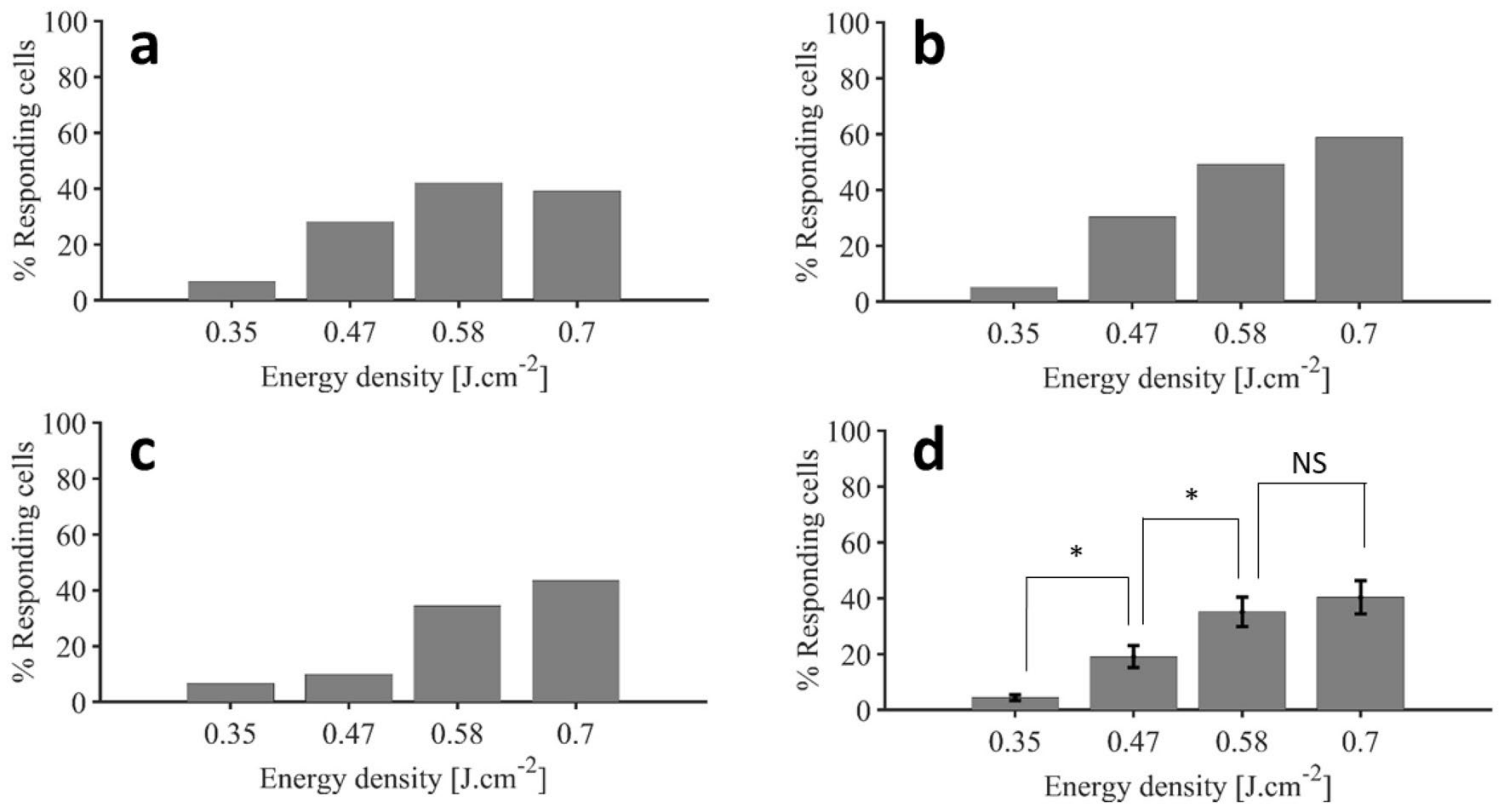
Percentage of cells exhibiting a clear calcium response threshold (**a**–**c**) Representative response distribution for varying IR energy densities respectively for M3, M3b and M4. (**d**) Average ± SEM of the percentages of cells exhibiting calcium response above the threshold determined for infrared exposure (two-sample t-test; * p < 0.05, NS p = 0.286; n = 5 experimental datasets from n = 4 mice) (figure created from Matlab R2018b).

Figure 3 shows straight ahead that infrared pulses of 0.35 J cm^−2^ induced calcium transients that could generate a fluorescence signal above noise in a very small population of cells. By gradually increasing the pulse energy density, intracellular calcium signals were evoked in an increasing number of cells. These effects are represented quantitatively in Fig. 4.

In order to confirm those observations and to have a better statistical view on the proportion of cells that responded or not, we first determined the signal-to-noise ratio (SNR) above which we could consider that the response was evoked (see “Methods”). The threshold was calculated for all ROIs separately to be sure that we only considered real calcium responses and to avoid any jitter effect, i.e. any noise from movement artefacts, breathing, heart rate pulsation, or photonic shot noise. Also, to ensure the robustness of our results, we made measurements on different areas (5 repetitions/area) from 4 animals. Due to the oblique angle of the mouse head and craniotomy under the objective according to the optical axis, the different imaging planes were in a range of 45 µm till 448 µm in the five experimental sessions (minimum depth: 100.84 ± 25.39 µm; maximum depth: 262.09 ± 49.49 µm; mean ± SEM; see Table 1 for individual values).

**Table 1:** Total number of cells identified in the fields of view and depth of the fields of view for each experiment considered in the analysis of this study.

Mouse	Mouse 1	Mouse 2	Mouse 3	Mouse 3 second area	Mouse 4
Total number of cells	51	136	294	228	212
Depth range of the field of view	45–175 µm	195–448 µm	96–255 µm	72–181 µm	96–251 µm

Figure 4 shows the percentage of responding cells per energy density, in three different mice as well as the average over the 5 experiments conducted in different mice. The three examples taken into account in the rest of this paper are the three experiments with the biggest number of identified cells in the field of view, i.e. Mouse 3, Mouse 3 second area and Mouse 4 (labeled respectively hereafter M3, M3b and M4).

Only a small number of cells (4.4 ± 1.1%, mean ± SEM, n = 5 experiments from N = 4 mice) responded to the 0.35 J cm^−2^ stimulus, suggesting that this energy density might be considered as non-stimulating for a working stimulation regime. Increasing the energy density to 0.47 J cm^−2^ caused a significant increase in the number of responsive cells (to 19.0 ± 4.4%, two-sample t-test, p = 0.012), indicating the existence of a stimulation energy threshold, below which cells don’t respond. Importantly, a further increase in the stimulus intensity to 0.58 J cm^−2^ causes another significant rise in the number of responding cells (35.1 ± 5.9%, t-test, p = 0.033), indicating a dose–response of this stimulus. Finally, an increase of the stimulus intensity from 0.58 to 0.70 J cm^−2^ did not recruit significantly more cells (40.3 ± 6.6%, t-test, p = 0.286), indicating a saturation of the stimulation energy–response level relationship. In summary: increasing the energy density delivered to the brains of mice resulted in a significant increase in the number of cells exhibiting calcium signals above our threshold in a reproducible and gradual way across different mice.

**Figure 5:**
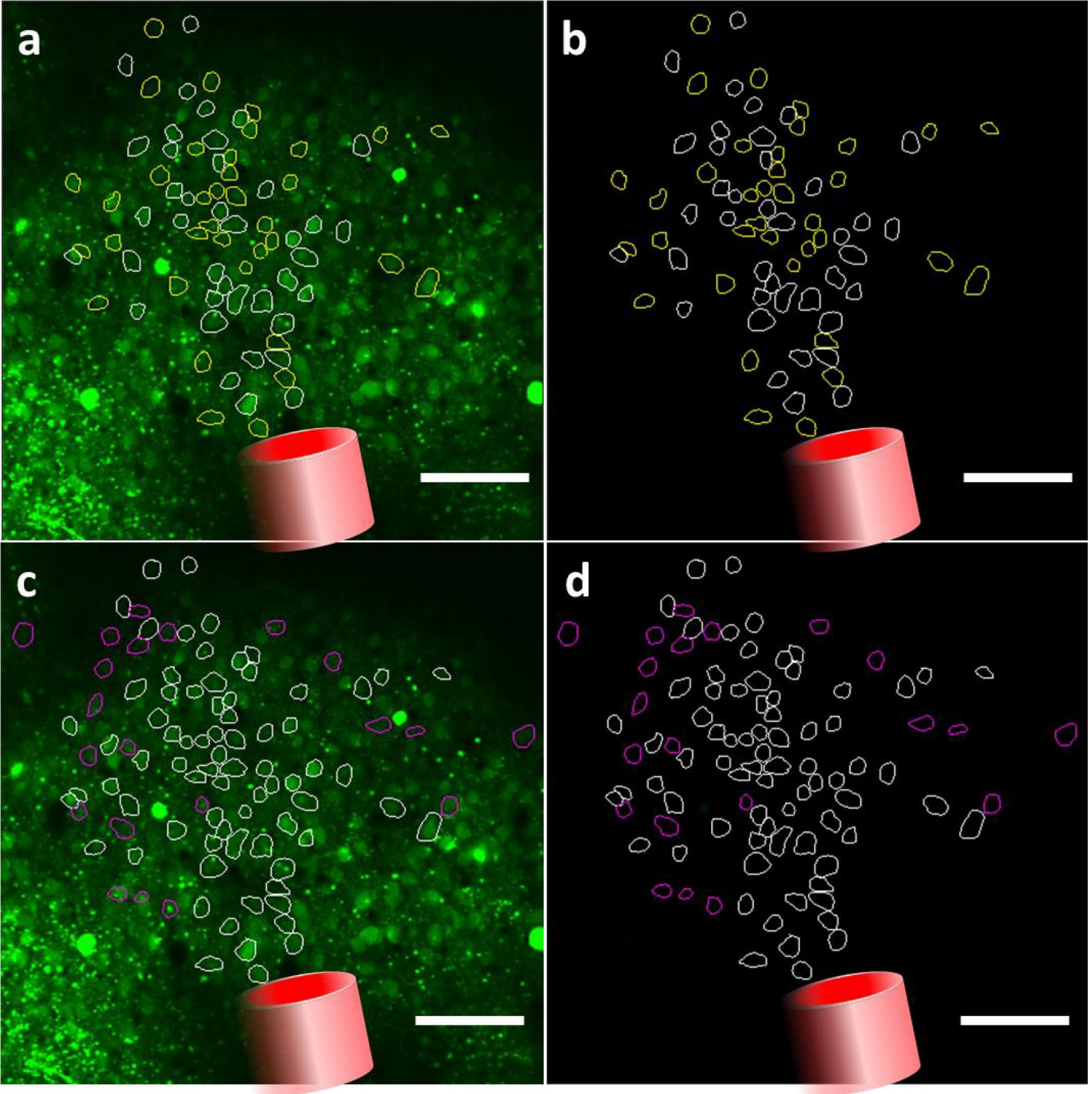
Representative two-photon field of view of a single mouse visual cortex showing responsive identified cells (**a**,**b**) Cells responding to 0.47 J cm^−2^, 0.58 J cm^−2^ and 0.70 J cm^−2^ in white and responding to 0.58 J cm^−2^ and 0.70 J cm^−2^ in yellow. (**c**,**d**) Cells identified in (**a**). in white and cells responding to only 0.70 J cm^−2^ in magenta. (**a**–**d**) Red cylinder represents the schematic view of the projection of the optical fiber transferring infrared light to the tissue. Scale bar: 100 µm (figure created from Powerpoint 2016 and MESc software).

As the number of responding cells differed for each energy condition, we can assume that the cells active in low energy conditions were not necessarily an overlapping population with those in high energy conditions. Therefore, we were interested in identifying the cells that were responding to the different stimulus intensities vs. cells that were not. Figure 5a,b (b same as Fig. 5a with an adjusted lookup table) shows the projection of the fiber tip orientation, as well as the position of the cells responding to three stimuli (*white*, (0.47 J cm^−2^, 0.58 J cm^−2^ and 0.70 J cm^−2^) and to only the two highest intensities of stimuli (*yellow*, 0.58 J cm^−2^ and 0.70 J cm^−2^). We do not show the lowest (0.35 J cm^−2^) stimulus in this example, as only very few cells responded to this stimulus intensity. Figure 5c,d show those two groups in white and the position of the cells only responding to the highest stimulus in magenta (0.70 J cm^−2^).

In Fig. 5a,b, one can see an example of 39 cells responding to the three stimuli (white) and 36 cells responding only to the two highest intensities of stimulation (yellow), as located in a similar area of the field of view in the infrared beam path, in agreement with the direction and orientation of the fiber tip observed in the upper z projection. Interestingly, if one considers the latter cells together (white Fig. 5c,d) and with respect to the 21 cells responding only to the highest stimulus (0.70 J cm^−2^), we can see that cells located more in the periphery of the cell distribution responded to multiple stimuli compared to those central to the beam path. As the infrared stimulation relies on a photothermal mechanism, a reasonable explanation of this observation might be that by increasing the energy density, the thermal stimulation threshold is achieved over a larger area. Indeed, the spatial distribution of the beam is Gaussian, meaning that the sides concentrate less energy leading to less thermal increase. Consequently, increasing the energy density probably implies that the thermal threshold necessary for the stimulation is achieved over a larger spatial area.

### Spatial extent of evoked responses

**Figure 6:**
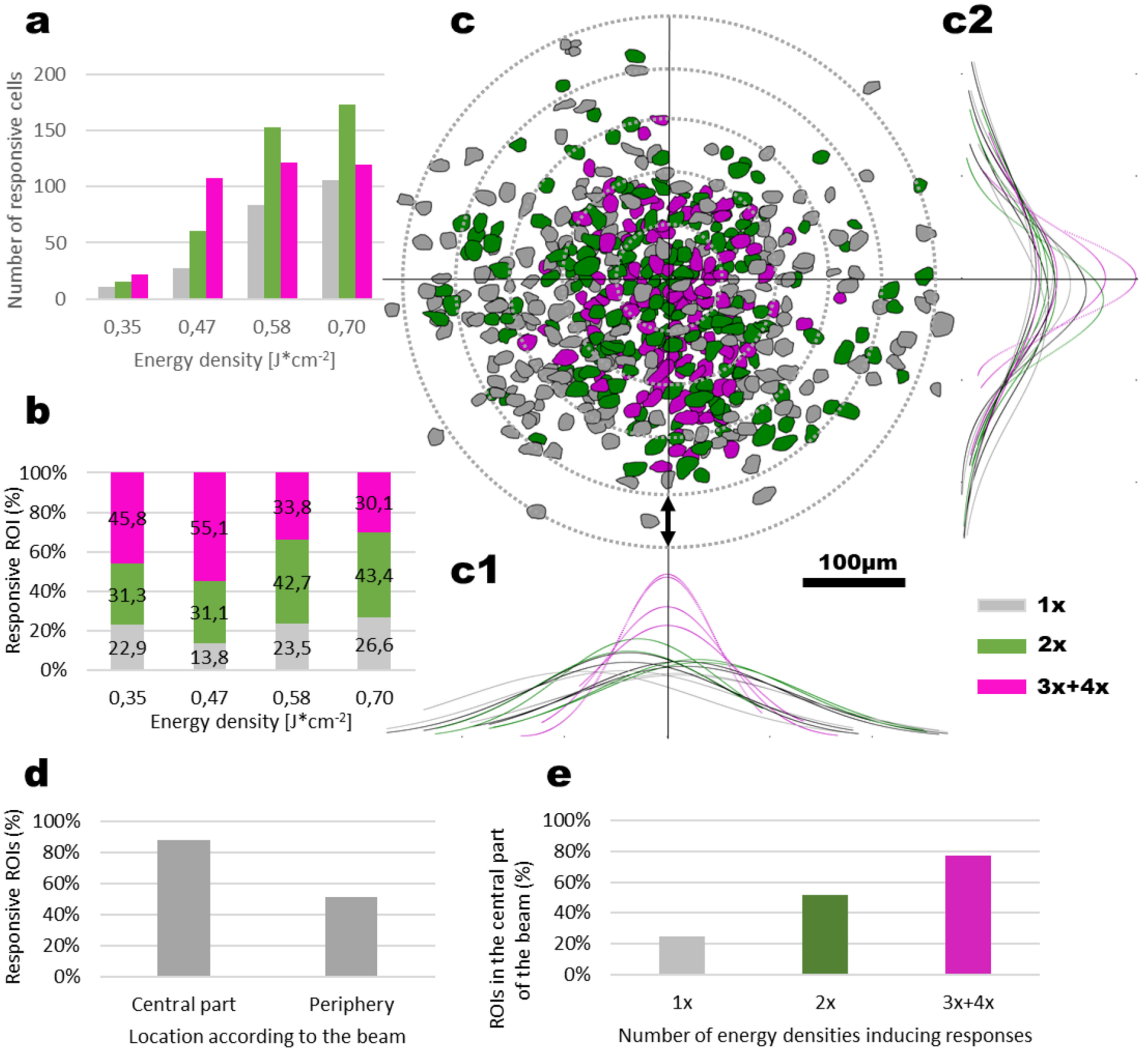
Spatial distribution of stimulation and cell recruitment pattern. (**a**,**b**) Number (**a** mean ± SEM) and percentage (**b**) of cells responding to a single (grey), two (green), or multiple (pink) stimulation intensities. (**c**) Summary overlay for all cells from 3 mice and 4 experiments, aligned to a central point marked by the maximal distribution probability for multiple responders both for the X (**c1**) and the Y (**c2**) axis. (**c1**,**c2**) Normal distribution fits to cells responding to single (1x, grey, n = 228 cells), double (2x, green, n = 402 cells) and multiple (3x + 4x, magenta, n = 371 cells) levels of energy density. Scale bar: 100 µm, Vertical double-headed arrow on (**c**): 50 µm. (**d**) Percentage of cells responding to at least one energy density stimulus in the central part of the beam compared to its periphery. (**e**) Percentage of cells responding to one, two and three or four energy densities located in the central part of the beam path (figure created from Excel 2016).

In order to acquire a more concise picture of the stimulated area, we superimposed one onto the other all experiments comprising all ROIs that have shown a response. We have separated the ROIs that responded to either one (n = 228 cells), two (n = 402 cells), or multiple (three and four; n = 371 cells) levels of stimulus energy density (Fig. 6). Figure 6a report the number of cells responding per energy density and were differentiated according to the number of other energy density stimuli that they responded to. To give an example in Fig. 6a, for the energy density 0.35 J cm^−2^, the sum of grey, green and pink bars is the total number of cells responding at 0.35 J cm^−2^. The grey bar is the number of cells that respond only to 1 energy density stimulus, i.e. in the present example to 0.35 J cm^−2^. The green bar represents the number of cells responding to 0.35 J cm^−2^, but also responding to another energy density stimulus (so 2×  for two stimulus energy densities inducing a response), and the pink bar represents the cells stimulated by 0.35 J cm^−2^, but also by two or the three others energy densities. Figure 6b follows the same logic, but is expressed as the percentage of all cells in the field of view per energy density. In order to compare across different experiments, we have centered the experimental field-of-view (FoV) from all animals to the spatial distribution maxima of multiple (3x + 4x) responders in X and Y of the field of view (Fig. 6c1,c2). The spatial distribution maximum is defined here as the peaks of the Gaussian repartition of multiple (3x + 4x) responders. The misalignments between each mouse FoV and each spatial distribution maxima was found to be matching the slight variations of the positioning of the optical fiber in the FoV. We could apply such a geometric shift as we have consistently placed the optical fiber at the same horizontal angle, so no FoV rotations were necessary for the overlay. By calculating the full width at half maxima for the spatial distribution of all the responsive cells, we could determine that the response could be evoked in an extending region. The cross-animal overlay shows the spatial extent of the response was 123.23 ± 12.70 µm from the center in X (range: 108.21 to 161.06 µm) and 95.12 ± 14.67 µm in Y (range: 62.67 to 132.94 µm). At the same time, we observed that cells that responded in the most robust way for almost all stimulation intensities were situated 66.25 ± 6.14 µm from the center in X (range: 56 to 82 µm) and 83.50 ± 15.95 µm in Y (range: 51 to 121 µm). This also makes comparisons possible for the imaged depth and for various fiber placement with respect to the FoV, as we only take into account the response, and not the stimulation source. Our results also show that in the center of the beam path, even lower energy levels can evoke consistent responses from cells that will also be activated with higher stimuli. Practically, these data point to the reliability and thus possible daily utility of this type of stimulation, with particularly stable and robust stimulation occurring in the center of the beam path (Fig. 6c). Furthermore, by defining the central part of the beam in Layer 2–3 as the ellipse having the size of the exposed surface of the brain to infrared (major axis of 246 µm and a minor axis of 168 µm), we found that 87.5% of the cells located in the center of the beam were responsive at least to one energy density applied, compared to 50.9% for outside of the central part of the beam path (Fig. 6d). Also, as it could be seen with the Gaussian repartition, there is a difference in the location of the cells responding to one, two and three or four energy densities: 77% of the cells responding to three/four energy densities are located in the central part of the beam path, against 51.8% of the cells responding to two energy densities and 24.8% of the cells responding only to one energy density (Fig. 6e). As we can see in Fig. 6a, most of the cells responding to one energy density are cells that exhibit a response at higher energy densities. This is coherent with Fig. 6e, as increasing the energy density induces an increase in the thermal effect on the sides of the beam (Gaussian shape) which therefore might pass from sub-stimulation threshold to supra-stimulation threshold.

### Energy density affects response amplitude

**Figure 7:**
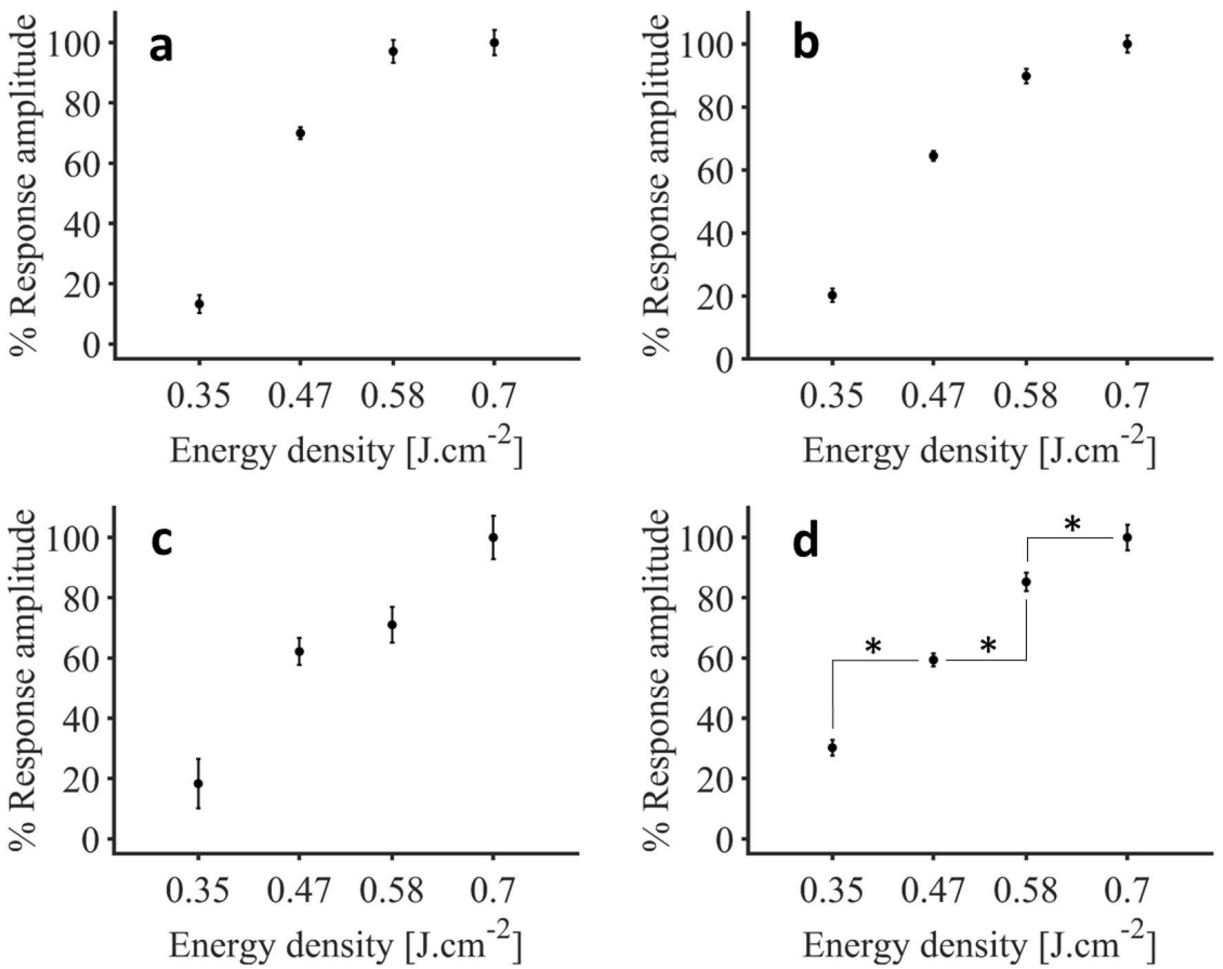
Representative dose–response relationship to IR energy densities for the cells not necessarily responsive to the lowest stimulus level (0.35 J cm^−2^), but responding to all stimuli above (0.47 J cm^−2^, 0.58 J cm^−2^ and 0.70 J cm^−2^), in percentage of the maximal response value. (**a**–**c**) Dose–response values respectively for M3, M3b and M4. (**d**) Comparison for responses of the cells responding to multiple stimuli (average ± SEM, n = 5 experiments from n = 4 mice; *p < 0.001) (figure created from Matlab R2018b).

Following the identification of responsive cells, we were interested in the impact of the energy density applied on the intensity of the infrared-evoked calcium signals. For that purpose, the peak values of the calcium signals were extracted for cells identified as responding at least to both 0.47 J cm^−2^, 0.58 J cm^−2^ and 0.70 J cm^−2^ and averaged for each mouse. Figure 7 shows the dose–response values obtained in three different mice as well as the average ± SEM over 5 experiments from 4 mice.

We previously pointed out that an energy density of 0.35 J cm^−2^ evoked a response only in a very low percentage of cells (4.4 ± 1.1% of all recorded cells). Thus, here, we looked at the population of cells that did not necessarily show any response to the lowest stimulus level (based on our previous threshold criteria, see “Methods”), but responded to all other stimuli (N = 98 cells from 4 animals and 5 experiments). In this population of highly responsive cells, we have taken the responses for the highest energy density of 0.70 J cm^−2^ as 100% (0.0729 ± 0.0020 ΔF/F_0_, 100% ± 2.77%, mean ± SEM). After verifying that this overall effect of the infrared stimulation was significant (p < 0.001, one-way repeated measures ANOVA), we looked at pairwise differences (post-hoc Bonferroni tests). When looking at a step lower from maximal stimulation, the response amplitude decreased significantly with the IR energy density of 0.58 J cm^−2^ (0.0654 ± 0.0017 ΔF/F_0_, 89.81 ± 2.27%, p < 0.001), just as with another step down to 0.47 J cm^−2^ (0.0470 ± 0.0011 ΔF/F_0_, 64.51% ± 1.55%, p < 0.001). Finally, we see that the baseline IR level of 0.35 J cm^−2^ calcium responses (0.0148 ± 0.0016 ΔF/F_0_) were also significantly different from the responses occurring at 0.47 J cm^−2^, p < 0.001). These results show that increasing IR stimulation is not just recruiting more cells, but also causing an increase in the activity of previously recruited cells, as there was evidence of increasing infrared-induced calcium signal amplitude with increasing energy density.

It should be noted that all experiments were performed on isoflurane-anesthetized animals, where it was previously shown that neuronal network activity and neuronal responsiveness is reduced when compared to awake network states^37,38^. Reduced neural network activity in cortical layer 2/3 was also reported in other types of anesthesia^39^. This might explain why we did not see any bursting activity triggered in cells, and that we did not have any significant change in the evoked calcium signals according to the energy density applied. Furthermore, based on previous calibration work with the same viral construct^40^, we can estimate that the calcium signals observed here correspond to the generation few action potentials which gives very weak differences in signal amplitude for the GCaMP6f calcium indicator used in this study. It is therefore difficult here to conclude any impact of infrared energy density on the magnitude of the calcium response amplitude.

### Assessment of tissue damage in the brain

**Figure 8:**
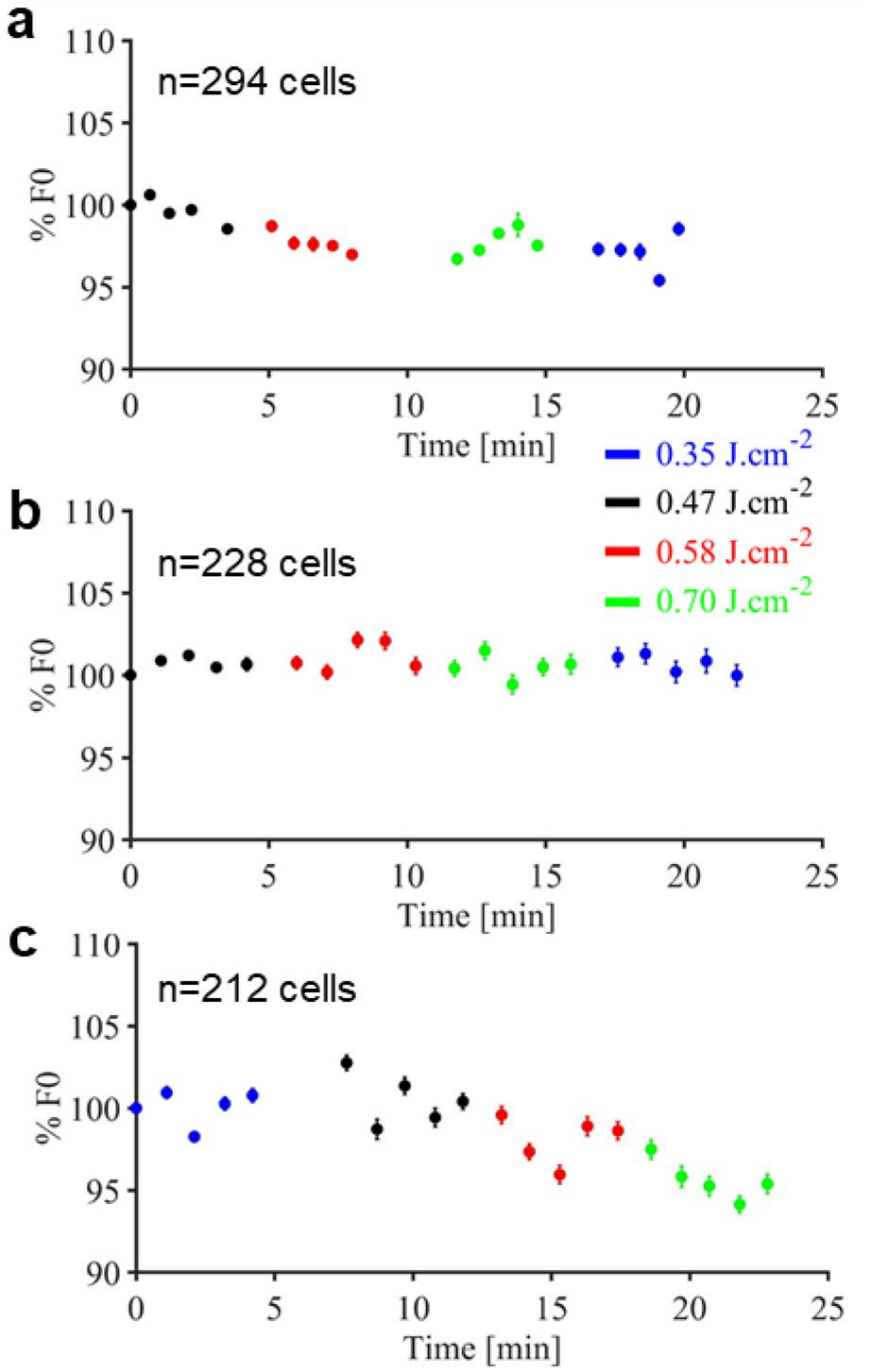
Representative baseline evolution over time respectively for M3, M3b and M4. (**a**–**c**) while applying different infrared exposure energy densities. Each point shows mean ± SEM for baseline fluorescence taken in a time window of 3 to 4.5 s and normalized with the baseline at the beginning of the experiments as reference (figure created from Matlab R2018b).

Infrared neural stimulation is mediated by a photothermal mechanism. Thus, the major concern of its application is the potential thermally induced damage that can occur. As an assessment of whether infrared stimulation was damage-free or not, all our recordings showed reproducibility in the calcium signals evoked by infrared exposure, and no baseline fluorescence increase was observed (see Fig. 8). The heat created by the infrared exposure may also affect blood vessels and cause bleeding in the brain. Such phenomenon would induce a significant drop in the two-photon imaging signals, which was not observed in any of the experiments, suggesting that no bleeding has occurred.

Figure 8 shows the evolution of the baseline (F_0_) over time and recordings. Figure 8a shows a stable baseline over time, indicating that no damage was done to the brain. In Fig. 8b,c, there is a slight decrease over time in the baseline, depending on whether we started the experiments by the lower or higher energy density. This decrease might be explained by the potential two-photon GCaMP bleaching as reported in the literature for long periods of recording^41^.

**Figure 9:**
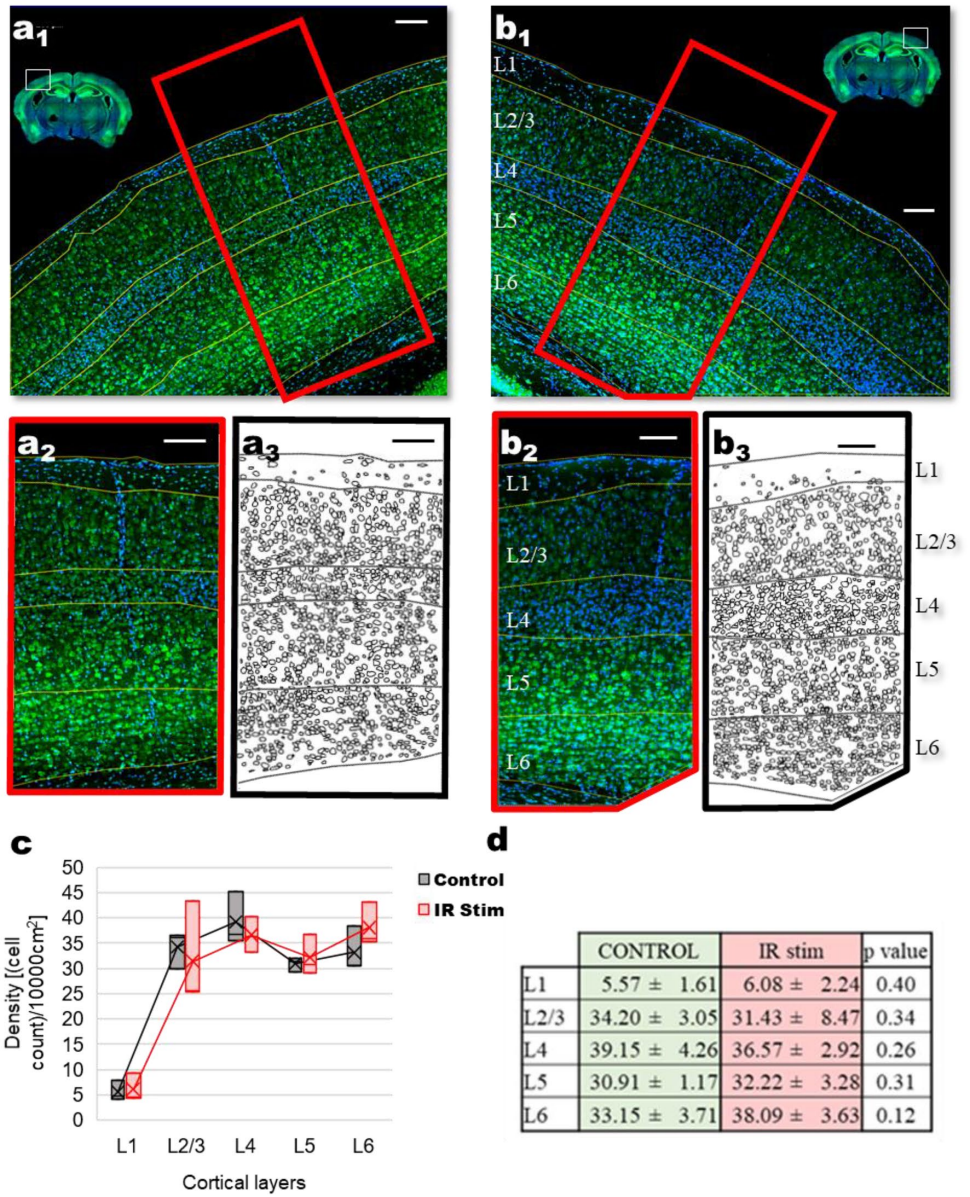
Confocal image of stained nuclei (DAPI staining; *blue*) and cell bodies (NeuroTrace green fluorescent Nissl staining, *green*) of (**a1**) control (non-irradiated) and (**b1**) infrared-irradiated mouse brain hemisphere. Control area (**a2**) corresponding to the (**b2**) localized IR-irradiated area, used for the detection of cell bodies. Identified cell bodies in (**a3**) control and (**b3**) irradiated area. (**c**) Box plot of cell density values per 0.1 mm^2^ for control and IR stimulation hemispheres. (**d**) Cellular density values (number of cells per 0.1 mm^2^; mean ± SD) for cortical layers in control and IR stimulation hemispheres, showing no significant difference (t-test, N = 3 mice). Scale bar: 100 µm (figure created from Excel 2016, Powerpoint 2016 and ImageJ).

A well-established method to asses tissue damage in the brain is with histological measurements^42^. In a separate set of experiments using intact cranial windows of Thy1-GCaMP6 transgenic mice to avoid the possible trace left by viral injection, we have set out to identify the possible cell loss that might follow after infrared stimulation (Fig. 9). We compared the area of the non-irradiated left hemisphere (Fig. 9a1,a2) to the right hemisphere, corresponding to the IR-stimulation site (Fig. 9b1,b2). As a positive control, a two-way ANOVA test showed significant layer differences in the layer-specific density of the identified cell bodies (F = 46.81547, p = 5.37668E−11), Fig. 9a3,b3), but no difference between the irradiated and non-irradiated hemisphere for the whole cortical column (F = 0.02748, p = 0.86972). Since lower cortical layers might compensate for any difference happening in cell density at layer 1 or 2/3, we have looked at layer to layer differences. As shown on Fig. 9c,d, out data and statistical analysis (two-sample t-test) showed no significant differences in cell densities between the control and infrared stimulated layers. These results suggest that no significant change in cell number can be attributed to the infrared stimulation.

## Discussion

In this paper, we showed that two-photon calcium imaging of mice expressing GCaMP6f in neurons is a useful tool to assess infrared-induced calcium signals in cortical layer 2/3 neurons in vivo. Infrared pulses of 250 μs repeated 100 times at 200 Hz elicited reproducible and calcium signals in single cells without any immediate apparent damage. By applying a threshold of three times the standard deviation of the noise to select cells responding to the infrared stimulus, we showed that increasing the energy density delivered to the mouse brain induced an increased number of responding cells, as well as an increase in response amplitude. The repeatability of the neural response to INS was shown by performing each experiment five times. Furthermore, by identifying the locations of the cells responding to one or multiple stimuli, we showed that cells responding to all energy levels or only to the higher ones were located in a well-defined area in the beam path, defined with respect to the orientation of the fiber tip. Cells responding to multiple, especially low energy, stimulations were situated more in the center of the area illuminated by the fiber, whereas cells responding only to the highest stimulus, i.e. 0.70 J cm^−2^, were preferentially located in its periphery. This might be the direct result of the fact that the thermal threshold necessary to reach infrared-induced calcium signals is obtained in a larger area while increasing the energy density, because of the Gaussian distribution of the light beam or to temperature diffusion. However, to fully confirm this point, investigating and mapping the temperature increase in vivo would be necessary. This could be done, e.g., by measuring INS-generated temperature gradients in vivo using either fluorescent dyes with temperature sensitive quantum yield^43^, as used in vitro, nanoparticles^44,45^, or newly available ratiometric temperature dyes^46^. This kind of study would also benefit from the elaboration of a consistent modelling study as it was done in other tissues^47^.

We chose to use the same temporal parameters for infrared stimulation (250 μs pulses, 200 Hz for 500 ms) as Cayce and colleagues have used in rodent brain^34^, with a similar range for energy density. However, few points differ between their study and the present manuscript. First, the wavelengths used are different: 1470 nm here compared to 1875 nm in the work of Cayce and colleagues. According to the available data of Hale and Querry^48^ reporting the water extinction coefficient k(λ), we interpolated the data to find the Lambert absorption coefficient of both wavelengths and found α(1470 nm) = 24,815 cm^−1^ and α(1875 nm) = 26,875 cm^−1^. This corresponds to penetration depths (calculated as the depth at which the infrared intensity is 37% of the initial intensity) of 400 microns and 370 microns respectively. Thus, despite the wavelength difference, it appears that both of them seem to be suitable for stimulating the first three layers of mouse cortices. Then there is also a difference in the imaging method, as in this work we image down to Layer 2/3 while in Cayce and colleagues work, the imaging was done at the surface of the brain (through 1 photon imaging) up to 100 µm deep (Layer 1, with 2 photon imaging). This difference in the depth of imaging is important, as the light was not focused and Layer 1 is experiencing higher irradiances and consequently higher temperature rises than Layer 2/3. This leads to the important point that not using focused light requires consideration regarding the damage threshold determination, as damage is expected to happen first where higher irradiances are, i.e. above the imaging plane used in the present study. Using focused light in the future would probably allow one to have a better window of safe stimulation if the target is Layer 2/3.

The calcium indicator used is also different, Oregon Green 488 BAPTA-1 AM in the case of Cayce and colleagues and GCaMP6f using a neuron specific promoter in the present study. The use of genetically encoded calcium sensors as GCaMP6f allows the perspective to implement chronic calcium imaging experiments without the need of dye injection prior to each imaging session. The use of this non cell type specific Oregon Green 488 BAPTA-1 AM indicator allowed Cayce and colleagues to identify two dynamics in the calcium signals observed: a fast response attributed to neuronal responses and a longer one attributed to glial cells through pharmacology. The fast dynamics that we report here are in the same range as the fast dynamics reported by Cayce and colleagues, a point that is discussed later in this discussion part. All of this leads also to the fact that the activation map made by Cayce and coworkers appeared to be larger than ours. On one hand, the fact that they labeled neuronal and glial populations, imaged in Layer 1, had a bigger fiber tip diameter (400 µm) than in our work (105 µm) gives reasons in the differences observed. On the other hand, they performed imaging in a bigger field of view than the one we could achieve here. Although they showed an extended activation map through calcium wave propagation in glial cells, from the data presented here, we can’t reject the hypothesis to have a bigger activation map than we can report with a slower diffusion as they were able to show. We showed single neuronal Ca^2+^ responses to INS in this study using mice expressing GCaMP6f through a neuron-specific promoter^49^. However, it is likely that astrocytes were also exposed to infrared light and it that Ca^2+^ signals were elicited through glial signaling as they reported. Thus, in future work, the interaction of infrared-induced glial calcium signals and infrared induced neuronal calcium signals could be studied with the use of calcium dyes or genetically encoded calcium indicators that can also label networks of astrocytes. Combining the non cell specificity of INS with two-photon imaging and cell specific calcium indicators might be very useful in the future in order to study neuron-glial cells interaction in vivo and the source of the calcium waves observed in the past.

It should be noted that the use of infrared exposure as a way of stimulation might have an effect on GCaMP6f itself. Indeed, it has been shown previously that temperature shifts have different effects on calcium sensors in general. First, increasing the temperature decreases the dissociation constant (Kd) of calcium sensors and chelators and this has been studied in the past in the case of GCaMP6f^50^. Second, temperature increase is known to negatively affect the quantum yield of the fluorophore and thus the fluorescence intensity, which might moreover differ between unbounded and bounded state of the calcium indicator^51^. Altogether, it is therefore likely that the strength of the calcium signals observed here is underestimated. However, without proper calibration of the temperature dependency of GCaMP6f at various calcium concentrations and the knowledge of the temperature increase reached in vivo with the infrared parameters used at the exact location of the calcium signals recorded, it is not possible to have an accurate correction of those signals. It should also be noted that all of our experiments were conducted on isoflurane-anesthetized animals, where it has been previously shown that neuronal network activity and neuron responsiveness is reduced when compared to awake network states^37,38^. Thus, further studies in awake animals will be essential to compare with our present work and to investigate the effect of INS on neuronal burst activity.

In this paper, infrared-induced calcium signals appeared to occur immediately at the onset of and only during the period of IR light illumination. However, according to the sampling rate of our experiments and the weak signal to noise ratio of the signals reported here, the estimation of the onset and time to peak durations is not possible to obtain with a good accuracy. It should be also taken into consideration that the time dynamics of calcium indicators depend on factors related to the indicators themselves but also to the intracellular calcium ion concentration that is reported: with higher concentration, latencies are longer. Hence, due to the low amplitude signals observed in this manuscript, the dynamics here are very fast and their estimation less accurate due to the acquisition set at ~ 31 frames per seconds, and low signal to noise ratio. In the case of GCaMP6f, a previous study on the impact on fluorescence properties of the reported signals according to the number of action potentials, and therefore on the calcium concentration reported, clearly show the impact of those parameters on the dynamics^52^. Then regarding dynamics reported in literature, either in vitro or in vivo, there was a delay between the onset of infrared light illumination and the calcium signals, which also lasted for a certain period of time after the termination of infrared light illumination. The comparison with those studies should take under consideration different points. First, calcium dynamics themselves differ according to the temperature. Calcium signals are indeed faster at physiological temperatures than for lower ones. Second, calcium dynamics in neurons are also faster than calcium dynamics in non excitable cells, as glial cells, as shown for example in Cayce and colleagues work^34^ with infrared stimulation where they identified two dynamics: a fast response attributed to neuronal responses and a longer one attributed to glial cells. Third, as the INS mechanism remain unclear despite the increasing knowledge of the scientific community over the years, the different temporal parameters used result in a difference in the temperature dynamics which might induce different calcium dynamic responses. Based on all of those points, the best choice to make a comparison appear to be using Cayce and colleagues work as a reference. By doing so, the fast dynamics that we report are in the same range than the fast dynamics reported by Cayce and colleagues. This suggests that here, we report only neuronal signals, which is coherent according to the transfection vector used AAV9.Syn.GCaMP6f.WPRE.SV40, based on the synapsin promoter which has been showed to be highly neuronal specific^49^. Furthermore, with the precision we could reach, we could not identify any difference in the latencies of responses between the central part of the beam and in its periphery. Altogether, it is consistent with the fact that in the same work they identified a calcium wave propagating in glial cells, but no propagation could be observed in axons and dendrites neuropil activity of Layer 1.

It has been reported in the literature that infrared-induced calcium signals might occur without action potentials, as shown in glial cells for instance^53^, and without extracellular calcium ions as it might be mainly mediated by internal calcium store release, from for instance the endoplasmic reticulum^18,20^. Determining the source of calcium signals in vivo would not be easy, as in vivo models are less suited for such pharmacology than cell cultures or brain slices. However, if the source is not defined, the increase of intracellular calcium ions concentration will induce, in physiological conditions for neurons, action potentials by depolarizing the membrane to the threshold. Furthermore, it is established that with two-photon calcium imaging and the sensitivity of the technology available to us (indicators, detectors…), it is not possible to detect subthreshold intracellular calcium increase in neurons^54^. Thus, it leads to the established fact that when doing two-photon calcium imaging of neurons in vivo, there is an inference between the calcium transients observed and the electrical activity of the neuron. As discussed previously, we assume that the signals reported here are neuronal signals and the low amplitude signals and short dynamics observed here are consistent with the fact that only a few action potentials are triggered^40,52^. In order to correlate infrared-induced calcium signals with the electrical activity, and have a better view on the whole electrochemical response of the brain to infrared stimulation, further work is needed, combining imaging with for instance implantation of transparent, flexible and biocompatible microelectrode arrays^55^.

Our histological assessment did not show any immediate apparent damage. However, the samples for histology were taken right after the imaging sessions (within less than an hour), in order to assess any immediate damage to the cells. Thus, in the interpretation of the histological images, it is important to notice that any changes that may have been caused by apoptosis and necrotic degeneration are not expected to be observable in this study at this rate of sampling. However, we do not see qualitative evidence of cell or tissue swelling, fragmented, lysed or ablated cells or displaced tissue. In the literature, histological studies after INS protocols have been performed. Chernov and colleagues^42^ showed evidence of tissue lesions from 0.4 J cm^−2^ in the rat brain in squirrel monkeys from 0.5 J cm^−2^. If those values are in the range of the ones used in our study, it should be taken into consideration that the pulse train used in the work of Chernov and colleagues (similar to ours) is applied each 5 s for a period of 30 min and over a larger area as the fiber tip was 200 microns diameter and with a wavelength (1875 nm), which is slightly more absorbed. Cayce and colleagues, in human spinal nerve^56^, highlighted that thermal damage was first noted at 1.09 J cm^−2^. However, any comparison is difficult to make as the temporal parameters of the stimulation differ (2 Hz stimulation for 10 s in their work) as well as the biological sample targeted. As another example, Goyal and coworkers^57^ investigated as well the safety of INS in guinea pig cochlea. After up to 5 h of stimulation, histology did not show a loss of spiral ganglion cells, hair cells, or other soft tissue structures of the organ. Light microscopy did not reveal any structural changes in the soft tissue either. However, they noticed a functional loss for pulse energies above 25 µJ, possibly a sign of early damage. In our work the energy per pulse varied between ~ 113.75 and ~ 227.50 µJ, but here again in an other tissue and during a less important duration. Altogether, we could not observe any functional loss in our dataset and no histological evidence of immediate damage appeared in our study. The present histological analysis reveals no immediate damage after a total of 20 stimulation protocols repeated every 30 s at the most. We can’t although ignore the possibility that, by applying this set of parameters more often and over a longer period of time would result in a higher probability of inducing stress and damage to the exposed tissue.

The present study was performed by delivering infrared light through a cranial window in anaesthetized mice experiments. However, the potential of using INS for the non-invasive cortical stimulation through the scalp is limited due to the absorption and scattering of infrared light through skin and bone. The precise spatial control of cortical neurostimulation with respect to the focal response location permitted by INS, could find clinical application in functional cortical stimulation. Indeed, during surgical procedures for epilepsy or tumor resection, the skull and dura are removed for locating lesions^58^. In this case, INS may provide a superior means of functionally stimulating and mapping the eloquent cortex with greater precision and with less risk than electrical neurostimulation by the delivery of current, due to lack of current spreading and in less invasive way, as it does not require any electrode insertion. In that sense, the present study shows that two-photon microscopy represent a remarkable tool in refining the experimental protocols and devices for INS, as well as further investigating in vivo interactions between cell types. Although the present work focuses on infrared stimulation, infrared inhibition might also benefit from two-photon microscopy through the use of Potassium indicators, as for instance the new series of GEPIIs^59^ (genetically encoded potassium ion indicators) which have been developed based on FRET technology that allow tracking of potassium concentration in vivo.

In conclusion, we have demonstrated the feasible application of INS in an in vivo setting for non-invasive neuronal stimulation using two-photon microscopy for the detection of activity. Since INS requires no preliminary labeling or markers, our work is a step towards non-invasive applications for brain mapping and a possible approach in replacing invasive therapeutic approaches in other neurological disorders, including in human clinical applications.

## Methods

### Experimental animals

All experimental protocols were approved by the Animal Care and Experimentation Committee of the Institute of Experimental Medicine of the Hungarian Academy of Sciences (approval reference numbers PEI/001/194-4/2014 and PEI/001/1771-2/2015). All procedures complied with Hungarian, French and European regulations for animal research and were carried out in compliance with the ARRIVE guidelines. All the procedures were performed on C57BI/6J (N = 5 males, RRID:IMSR_JAX:000664) or Thy-1-Cre (N = 10 males, RRID: IMSR_JAX:006143_JAX:010908) mice (P60-120). Animals had to food and water ad libitum and were maintained in temperature-, humidity-, and light-controlled conditions.

### AAV labeling

The injection procedure was performed as described previously^52^, adjusted with some minor modifications. A hole of about 0.5 mm diameter was opened in the skull with the tip of a dental drill over the V1 cortical region (centered 2.5 mm lateral and 3.0 mm posterior to the bregma). Injections were made with a glass micro-pipette (tip diameter ≈ 10 µm) which were back-filled with 0.4 µl vector solution (≈ 6 × 10^13^ particles/ml). The solution was then injected slowly (10 nl/s for first 30 nl, and 0.5 nl/s for the remaining quantity) into the cortex, at a depth of 450 µm under the pia. For population imaging, we used AAV9.Syn.GCaMP6f.WPRE.SV40 or AAV9.Syn.Flex.GCaMP6f.WPRE.SV40 (in the case of Thy-1-Cre); both viruses were from Penn Vector Core, Philadelphia, PA and are neuron-specific^52^. The cranial window was implanted over the injection site 2 weeks after the injection, as described in the surgical procedure section.

### Surgical procedure

The surgical procedure was similar to the one described previously^60^, adjusted with some minor modifications. As a summary, mice were anesthetized with a mixture of midazolam, fentanyl, and medetomidine (5 mg, 0.05 mg and 0.5 mg/kg body weight, respectively). Finally, a circular craniotomy was made over the V1 region using a dental drill, and was fully covered with a cover glass.

### Optical fiber preparation

A multimode laser diode (LU1470T015, Lumics) with a fibered output (core diameter 105 μm, numerical aperture 0.15) was used to provide 1470 nm infrared radiation. The module temperature was controlled using the appropriate cooling block (LU_CB_T_0, Lumics). The optical fiber was placed into a custom-built guide cannula and mounted onto the arm of a micromanipulator (LN Junior LR, Luigs-Neumann, Ratingen, Germany).

### Infrared light stimulation

The power supply (LU_DR_AD, Lumics) allowed control of both the exposure time and the power of the infrared light delivered to the biological tissue. It was externally controlled by a waveform generator (3390 waveform generator, Keithley) triggered by MESc acquisition software. Average power was measured using a S145C integrating sphere photodiode power sensor attached to the PM100D power meter (Thorlabs) from the tip of the fiber after the completion of each experiment, to be sure that no damage was done to the tip of the fiber. Energy density of infrared pulses was calculated as the energy density delivered by the tip of the fiber to avoid any approximation in the calculation (diameter = 105 μm). The temporal profile of the infrared stimulation consisted of 250 μs pulses applied at 200 Hz during 500 ms.

Energy density applied during stimulation is expressed in this paper as the energy density deposited at the surface of the brain, in order to be consistent with the literature. To do so, multiple parameters were taken into consideration during the calculation of the area exposed to infrared: the fiber core diameter, its numerical aperture, the angle between the fiber and the optical axes and the angle between the mouse brain surface and the optical axis (both angles were measured with two-photon z-stacks), the width of the cranial window and the different refractive indexes (immersion gel, cranial window and brain tissue). After calculation, the surface of the brain exposed to infrared was found to be an ellipse with a big axis of 246 µm and a small axis of 168 µm representing a total area of about 3.25 × 10^–4^ cm^2^. It should be noted that the immersion gel contains 99% of water. Therefore, it is expected that this gel absorbs as well some infrared light. This absorption was not taken into account in the calculation of the energy density, due to the different angles, but should be taken into consideration.

### In vivo calcium imaging data acquisition

After a brief sedation by 2% isoflurane, the animal was placed on a heating pad to maintain its’ temperature at 38 °C, and its head was fixed by screwing the head-fixed metal bar to the custom mouse holder in the microscope setup. During the recording procedure, the anesthesia was maintained with a 0.5% isoflurane and carbogen mixture. An ultrasound transmission gel (AquaUltra Clear, Ultragel Hungary 200 Ltd, Hungary) was applied as an immersion medium. The mounted optical fiber was positioned underneath a 16× objective (Nikon LWD 16x/0.8 NA) under IR camera guidance. Switching to two-photon mode, a reference z-stack of the volume was acquired on a dual-scanhead two-photon microscope (FemtoS-Dual, Femtonics Ltd, Budapest, Hungary) equipped with a femtosecond pulsed laser tuned to 910 nm (Chameleon Ultra II, Coherent, Santa Clara, California). A single acquisition plane was selected and full-frame imaging was started in resonant scanning mode at 30.9375 Hz. The control of calcium signal recording and the trigger of infrared stimulation was done using the microscope’s acquisition software (MESc, Femtonics Ltd, Budapest, Hungary).

### Histological evaluation

The tissue was taken after the imaging sessions (within less than an hour) to assess any immediate damage and necrosis via counting Nissl-stained cells at the irradiated cortical column—a widely accepted method for studying cell loss^61^. Animals (N = 3) were transcardially perfused first with saline, then with 150 ml of fixative solution containing 4% PFA in 0.1 M phosphate buffer (PB). Tissue blocks were cut on a Vibratome (Leica VT1200S) into 50 µm coronal sections. After extensive washes in PB, Nissl stanning was used (1:200 in PB) (NeuroTrace 500/525 Green Fluorescent Nissl Stain, Thermo Fisher) followed by 2-(4-amidinophenyl)-1H -indole-6-carboxamidine (DAPI) (1:10,000 in PB). Sections were mounted on normal slides with gelatin coating solution and covered with mounting medium (Aquamount Mounting Medium with DAPI, Polysciences Inc.). Histological data were evaluated by confocal microscopy (Ni-EC2, Nikon and LSM 510, Zeiss Axioscope): after locating the site of infrared stimulation based on stereotactic coordinates using the Mouse Brain Atlas^62^, tile scans of both the control and the irradiated areas were taken (Fig. 9). Using a generalist algorithm for cellular segmentation^63^, neuronal cell bodies were identified in a 500 µm wide cortical column centered around the irradiated area, and also for the corresponding contralateral control hemisphere.

### Data analysis

Fluorescent time series depicting the calcium changes occurring in vivo in both the neuropil and the selected cells were analyzed with the MESc data acquisition software (Femtonics Ltd, Budapest, Hungary) and the MES curve analyzer tool. Briefly, regions of interest were manually selected to coincide with single cell somata. The raw fluorescence calcium traces were extracted to Excel and analyzed using custom Matlab scripts. They were transformed to show fluorescence change according to Eq. (1):

1 $$\frac{\Delta F}{{F_{0} }} = \frac{{\left( {F - F_{0} } \right)}}{{F_{0} }}$$

where *F* is the fluorescence at any given point in time, and *F*_*0*_ is the mean fluorescence value for the 3000 to 4500 ms range, preceding the infrared stimulus presentation. Following this, the signal-to-noise ratio (SNR) is:

2 $$SNR = {{\left( {\frac{\Delta F}{{F_{0} }}} \right)_{peak} } \mathord{\left/ {\vphantom {{\left( {\frac{\Delta F}{{F_{0} }}} \right)_{peak} } {\sigma F}}} \right. \kern-\nulldelimiterspace} {\sigma F}}$$

where σF is the standard deviation of the baseline period. We took those events as a Calcium response that exceeded the threshold of three times the standard deviation of the baseline period.

For each mouse, outliers were identified using the boxplot function of Matlab and were removed from the analysis. Normal distribution for the spatial extent of the response has been determined using standard Matlab functions. Statistical analysis was done using OriginPro.

### Disclosures

AK and IV were funded by ANR [TRAJECTORY] and recurrent funding from CNRS & Aix Marseille Université. RO and DM were funded by foundation EDF ATPulseGliome. GS AB and BR were funded by ERC 682426, VISONby3DSTIM. AS and BH were funded by ERC StG 715043. The authors are grateful to K. Lengyel for technical assistance. We acknowledge the help of the Nikon Microscopy Center at the Institute of Experimental Medicine, Budapest, Hungary. All experimental protocols were approved by the Animal Care and Experimentation Committee of the Institute of Experimental Medicine of the Hungarian Academy of Sciences (approval reference numbers PEI/001/194-4/2014 and PEI/001/1771-2/2015) and by the French Ministry of Higher Education, Research and Innovation (approval reference number APAFIS#22182–2019091818381928 v5). All procedures complied with Hungarian, French and European regulations for animal research.

## Supplementary Information

Supplementary Information 1.

Supplementary Video S1.

## Data Availability

The authors declare that the data supporting the findings of this study are available within the paper and its supplementary information files.

## Code availability

Matlab code used in this project for data analysis is available from the corresponding author upon reasonable request.
