# Supplementary material for: Two-photon GCaMP6f imaging of infrared neural stimulation evoked calcium signals in mouse cortical neurons in vivo
Source: Sci Rep. 2021 May 7;11:9775. doi: 10.1038/s41598-021-89163-x (PMC8105372; doi:10.1038/s41598-021-89163-x)
Supplement: Supplementary file 1 — Supplementary Information 1. [file 41598_2021_89163_MOESM1_ESM.pdf]

Two-photon GCaMP6f imaging of infrared neural stimulation evoked calcium signals in mouse cortical neurons *in vivo*

Attila Kaszas[1,2], Gergely Szalay[3], Andrea Slézia[1,4], Alexandra Bojdán[3], Ivo Vanzetta[2], Balázs Hangya[4], Balázs Rózsa[3,5], Rodney O'Connor[1,2], David Moreau[1,*]

[1]Mines Saint-Etienne, Centre CMP, Département BEL, F - 13541 Gardanne France

[2]Institut de Neurosciences de la Timone, CNRS UMR 7289 & Aix- Marseille Université 13005 Marseille, France

[3]Laboratory of 3D Functional Network and Dendritic Imaging, Institute of Experimental Medicine, Budapest 1083, Hungary

[4]Lendület Laboratory of Systems Neuroscience, Institute of Experimental Medicine, Budapest 1083, Hungary

[5]Two-Photon Laboratory, Faculty of Information Technology, Pázmány Péter Catholic University, Budapest 1083, Hungary

Supplementary movie S1.

Three-dimensional reconstruction from a two-photon z-stack of a single representative experiment. Green shows the GCaMP6f labeling, blue is the coverglass of the cranial window, and the optical fiber is depicted in red, placed above the cranial window. The box shows units in um.
